# Defining hypoxaemia from pulse oximeter measurements of oxygen saturation in well children at low altitude in Bangladesh: an observational study

**DOI:** 10.1136/bmjresp-2021-001023

**Published:** 2021-11-02

**Authors:** Eric D McCollum, Carina King, Salahuddin Ahmed, Abu A M Hanif, Arunangshu D Roy, ASMD Ashraful Islam, Tim Colbourn, Holly B Schuh, Amy Sarah Ginsburg, Shubhada Hooli, Nabidul H Chowdhury, Syed J R Rizvi, Nazma Begum, Abdullah H Baqui, William Checkley

**Affiliations:** 1 Global Program in Respiratory Sciences, Eudowood Division of Pediatric Respiratory Sciences, Department of Pediatrics, School of Medicine, Johns Hopkins University, Baltimore, Maryland, USA; 2 Health Systems Program, Department of International Health, Johns Hopkins Bloomberg School of Public Health, Baltimore, Maryland, USA; 3 Department of Global Public Health, Karolinska Institutet, Stockholm, Sweden; 4 Projahnmo Research Foundation, Sylhet, Bangladesh; 5 Global Health Institute, University College London, London, UK; 6 Department of Epidemiology, Johns Hopkins Bloomberg School of Public Health, Baltimore, Maryland, USA; 7 Clinical Trial Center, University of Washington, Seattle, Washington, USA; 8 Section of Emergency Medicine, Department of Pediatrics, Baylor College of Medicine, Houston, Texas, USA; 9 Division of Pulmonary and Critical Care, Department of Medicine, School of Medicine, Johns Hopkins University, Baltimore, Maryland, USA; 10 Center for Global Non-Communicable Disease Research and Training, School of Medicine, Johns Hopkins University, Baltimore, Maryland, USA

**Keywords:** respiratory infection, pneumonia, paediatric lung disaese

## Abstract

**Background:**

WHO defines hypoxaemia, a low peripheral arterial oxyhaemoglobin saturation (SpO_2_), as <90%. Although hypoxaemia is an important risk factor for mortality of children with respiratory infections, the optimal SpO_2_ threshold for defining hypoxaemia is uncertain in low-income and middle-income countries (LMICs). We derived a SpO_2_ threshold for hypoxaemia from well children in Bangladesh residing at low altitude.

**Methods:**

We prospectively enrolled well, children aged 3–35 months participating in a pneumococcal vaccine evaluation in Sylhet district, Bangladesh between June and August 2017. Trained health workers conducting community surveillance measured the SpO_2_ of children using a Masimo Rad-5 pulse oximeter with a wrap sensor. We used standard summary statistics to evaluate the SpO_2_ distribution, including whether the distribution differed by age or sex. We considered the 2.5th, 5th and 10th percentiles of SpO_2_ as possible lower thresholds for hypoxaemia.

**Results:**

Our primary analytical sample included 1470 children (mean age 18.6±9.5 months). Median SpO_2_ was 98% (IQR 96%–99%), and the 2.5th, 5th and 10th percentile SpO_2_ was 91%, 92% and 94%. No child had a SpO_2_ <90%. Children 3–11 months had a lower median SpO_2_ (97%) than 12–23 months (98%) and 24–35 months (98%) (p=0.039). The SpO_2_ distribution did not differ by sex (p=0.959).

**Conclusion:**

A SpO_2_ threshold for hypoxaemia derived from the 2.5th, 5th or 10th percentile of well children is higher than <90%. If a higher threshold than <90% is adopted into LMIC care algorithms then decision-making using SpO_2_ must also consider the child’s clinical status to minimise misclassification of well children as hypoxaemic. Younger children in lower altitude LMICs may require a different threshold for hypoxaemia than older children. Evaluating the mortality risk of sick children using higher SpO_2_ thresholds for hypoxaemia is a key next step.

**Key messages**

- The ideal peripheral arterial oxyhaemoglobin saturation (SpO_2_) threshold for defining hypoxaemia among children in low-income and middle-income countries is unknown.
- A SpO_2_ threshold for hypoxaemia set at any of the 2.5th, 5th or 10th percentiles of SpO_2_ measurements from well children in a lower altitude setting is higher than the <90% threshold currently recommended by the WHO.
- This study is a possible model for other research seeking to establish SpO_2_ thresholds for hypoxaemia in children and provides evidence for health policy makers to consider before implementing higher SpO_2_ thresholds than currently in practice in lower altitude settings of low-income and middle-income countries.

## Introduction

Lower respiratory infections (LRIs) kill more young children than any other infectious disease in the world.^1^ The most recent 2017 global estimates report more than 800 000 LRI deaths annually among children below 5 years of age,^1^ equating to 1–2 deaths every minute. The vast majority of paediatric LRI deaths occur in low-income and middle-income countries (LMICs).^1^ Approximately 30% of all global LRI deaths take place in South Asia each year, and Bangladesh has the third highest annual paediatric LRI incidence and mortality burden among all South Asian countries.^1^

LRIs may be complicated by pulmonary inflammation and areas of ventilation-perfusion mismatch that cause acute hypoxaemia, or a low peripheral arterial oxyhaemoglobin saturation (SpO_2_) as measured non-invasively by a pulse oximeter.^2^ Acute hypoxaemia is an important risk factor for mortality among children with LRIs in LMICs.^3^ For LMICs at lower altitude (ie, <2500 m) the SpO_2_ hypoxaemia threshold endorsed by WHO is <90%, a threshold associated with elevated mortality risk among children with LRIs like pneumonia.^3–5^ Per WHO guidelines children with caregiver reported cough and/or difficult breathing accompanied by a SpO_2_ <90% are recommended for hospitalisation, parenteral antibiotics and oxygen administration.^4 5^ Recent observational studies from Malawi reveal that SpO_2_ thresholds higher than 90% may also be associated with elevated mortality risk among children under 5 years with clinically diagnosed pneumonia.^6–8^ This evidence suggests the current WHO SpO_2_ hypoxaemia threshold of <90% may be suboptimal for identifying higher risk paediatric pneumonia cases for hospitalisation in some LMICs.

Despite both the importance and uncertainty around the optimal SpO_2_ threshold for defining hypoxaemia few studies from lower altitude settings in LMICs address this issue. One approach commonly used for deriving thresholds for diagnostic tests is to produce a reference range from a healthy population representative of the test’s intended target population.^9 10^ This approach has been applied to SpO_2_ measurements in children, with most research to date focused on children residing at higher altitudes.^11–15^ In this study, we define hypoxaemia from the SpO_2_ distribution of well children residing at lower altitude in rural Bangladesh who are participating in a pneumococcal conjugate vaccine (PCV) effectiveness study. We also consider the potential health system implications of implementing a SpO_2_ threshold for hypoxaemia derived from a population of well children.

## Methods

### Study design

This is a prospective observational study within a PCV effectiveness evaluation.^16^

### Study setting

Between June and August 2017, the Projahnmo research group, a collaboration between Johns Hopkins University, the Government of Bangladesh’s Ministry of Health and Family Welfare, and Bangladeshi non-governmental and academic institutions, conducted this substudy in three subdistricts (upazilas) of Syhlet district, northeast Bangladesh. The parent study took place between January 2014 and June 2018.^17^

**Figure 1:**
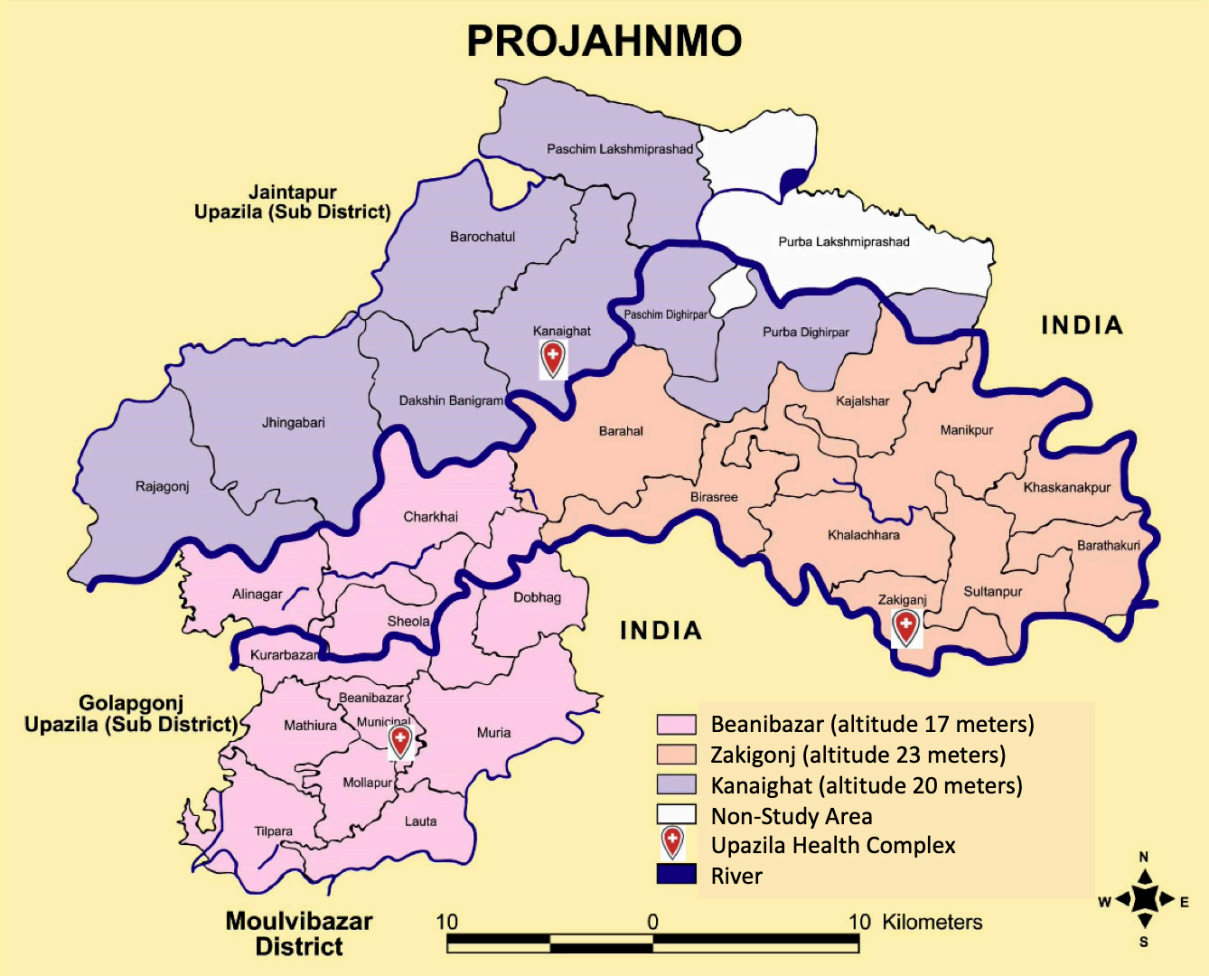
Projahnmo research foundation surveillance area.

The Projahnmo research group has a well-established community surveillance system.^17^ The three upazilas under routine surveillance, Zakiganj, Kanaighat and Beanibazar, are at an altitude between 17 and 23 m and have a total population of about 770 000 (figure 1). During routine surveillance local female residents called community health workers (CHWs) visit households within an area of about 10 000 population every 2 months. At each surveillance visit these trained CHWs provide health counselling to families regarding illness recognition and care seeking, screen women for pregnancy, and evaluate children for respiratory illnesses.

### Data collection

Twenty-two CHWs were trained in September 2015 to use a Masimo Rad-5 pulse oximeter with an LNCS Y-I wrap sensor as a part of enhanced respiratory surveillance activities for children during the parent PCV study. The initial training was 1 day and included theoretical sessions on pulse oximetry supplemented by practice using pulse oximeters to measure the SpO_2_ of volunteer adults and children. During the study period, CHWs participated in refresher sessions at least every 6 months and were routinely supervised by study physicians during household participant screening with the device. Remediation was provided when needed. CHWs were trained to apply the wrap sensor to the big toe of children and gently hold the foot to mitigate movement artefact. SpO_2_ values were considered adequate quality measurements when the CHW achieved the following three metrics; (1) the SpO_2_ value remained stable and non-drifting for no less than three seconds, (2) the quality index signal was of consistent amplitude and displayed at least three green bars and (3) the perfusion index signal was at least three green bars in amplitude.

Between June and August 2017, CHWs enrolled well children aged 3–35 months participating in surveillance. CHW screening included an examination for acute signs of an illness and asking caregivers whether the child had any symptoms during the prior week. CHWs observed children for cough, counted the child’s respiratory rate for 1 min, measured an axillary temperature with a thermometer, and observed children for any sign of respiratory distress (ie, head nodding, nasal flaring, audible wheezing, grunting, stridor, tracheal tugging or lower chest wall indrawing). Children were excluded and referred to the study clinic if aged 3–11 months and had a respiratory rate of >50 breaths/minute, or 12–35 months old with a respiratory rate of >40 breaths/min, an axillary temperature >38.8°C, any vomiting or diarrhoea, any WHO-defined general danger sign (lethargy, convulsions, not eating or drinking, severe acute malnutrition), or any sign of respiratory distress as specified above. Children with isolated nasal congestion and/or rhinorrhoea were not considered acutely ill and were enrolled.

In order to further filter potentially unwell children from our sample, post hoc we created three analytical samples from children with a recorded SpO_2_ measurement using different reference heart rate ranges, since an abnormal heart rate may suggest unrecognised illness. Analytical sample 1 is our primary analytical sample, and applies the most conservative estimate of ‘healthy’ with relatively narrow normal heart rate reference ranges of: 120–160 beats/min for 3–5 months, 110–150 beats/min for 6–11 months, 100–140 beats/min for 12–23 months and 90–130 beats/min for 24–35 months.^18^ Analytical sample 2 is less conservative as it has less restrictive heart rate reference ranges of 100–190 beats/min for 3–23 months and 60–140 beats/min for 24–35 months as normal reference ranges.^18^ Analytical sample 3 ignores heart rate reference ranges altogether and assumes all children are healthy.

### Statistical analysis

Normally distributed continuous variables were described using means and SD, non-normally distributed continuous variables were characterised by medians and IQRs, and bivariate or categorical variables were described using proportions. We considered the 2.5th, 5th and 10th percentile of SpO_2_ as possible thresholds for defining hypoxaemia. We used the Wilcoxon-Mann-Whitney test for comparisons including a dependent variable without a normal distribution. The Kruskal-Wallis test was used for comparisons between a multilevel independent variable and a dependent variable lacking a normal distribution. We fit a linear regression model, adjusted for sex, to explore the association between SpO_2_ and age. Using a power of 80%, significance level of 0.05, and that 25% of children will either be ill, unavailable or fail measurement, we needed to screen 700 households for each of the three child age strata of 3–11 months, 12–23 months and 24–35 months (total 2100) to estimate a mean SpO_2_ of 96%±0.2%. Stata V.16.0 was used for all analyses.

### Patient and public involvement

The development, design, recruitment, conduct and results of the parent PCV evaluation and this nested study were communicated to the public through local community sensitisation meetings held by the Projahnmo study group consortium in Sylhet, Bangladesh.

## Results

### Participant characteristics

**Figure 2:**
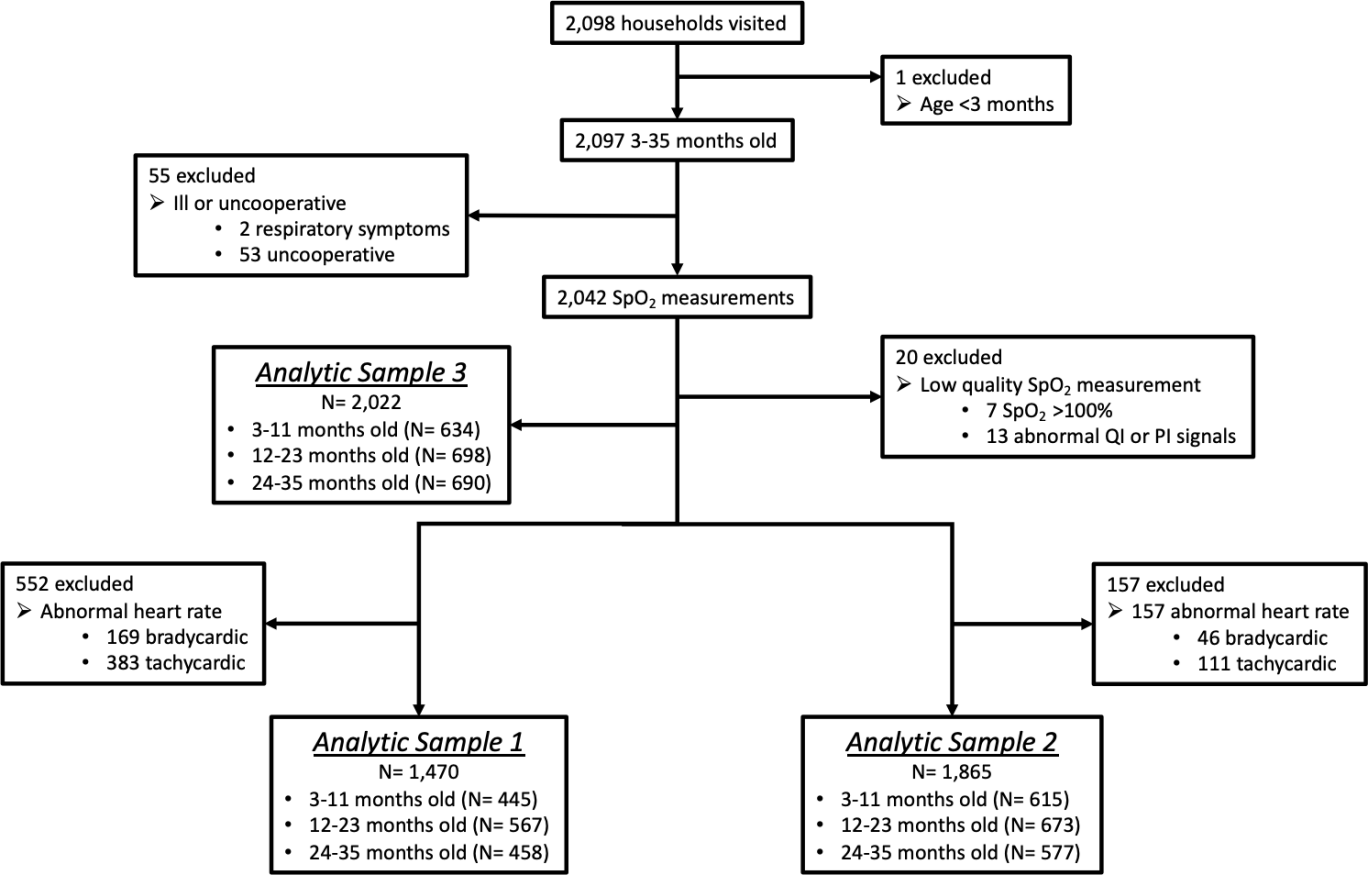
Study profile. SpO_2_, peripheral arterial oxyhaemoglobin saturation. PI, perfusion index; QI, quality index.

From June to August 2017, the CHWs visited 2098 households and attempted SpO_2_ measurements on 2042 children (figure 2). Overall, 20 children with low-quality SpO_2_ measurements were excluded at the analysis stage. For primary analytical sample 1, a total of 552 children were additionally omitted due to abnormal heart rates. For analytical sample 2, 157 children were excluded based on reference heart ranges. Among the 1470 children analysed for analytical sample 1, the mean age was 18.6 months (SD, 9.5) (table 1). Average age and the proportion of participants who were female were similar across the three analytical samples.

**Table 1:** Characteristics and SpO_2_ distribution of three analytical samples

Characteristic	Analytical sample 1 (primary)*				Analytical sample 2†				Analytical sample 3‡			
	All N=1470	3–11 months N=445	12–23 months N=567	24–35 months N=458	All N=1865	3–11 months N=615	12–23 months N=673	24–35 months N=577	All N=2022	3–11 months N=634	12–23 months N=698	24–35 months N=690
Age in months, mean (SD)	18.6 (9.5)	7.3 (2.4)	18.0 (3.3)	30.3 (3.2)	18.2 (9.6)	7.2 (2.5)	18.0 (3.3)	30.2 (3.2)	18.7 (9.7)	7.2 (2.5)	18.0 (3.3)	30.1 (3.2)
Females, n (%)	697 (47.4%)	203 (45.6%)	274 (48.3%)	220 (48.0%)	904 (48.4%)	293 (47.6%)	332 (49.3%)	279 (48.3%)	988 (48.8%)	302 (47.6%)	348 (49.8%)	338 (48.9%)
SpO_2_, 95th percentile	100%	100%	100%	100%	100%	100%	100%	100%	100%	100%	100%	100%
SpO_2_, 75th percentile	99%	99%	99%	99%	99%	99%	99%	99%	99%	99%	99%	99%
SpO_2_, 50th percentile	98%	97%	98%	98%	98%	97%	97%	98%	98%	97%	97%	98%
SpO_2_, 25th percentile	96%	96%	96%	96%	96%	96%	96%	96%	96%	96%	96%	96%
SpO_2_, 10th percentile	94%	94%	94%	95%	94%	94%	93%	94%	94%	94%	93%	94%
SpO_2_, fifth percentile	92%	93%	92%	93%	92%	92%	92%	92%	92%	92%	92%	92%
SpO_2_, 2.5th percentile	91%	92%	91%	91%	91%	91%	91%	91%	91%	91%	91%	92%
SpO_2_, range	90%–100%	90%–100%	90%–100%	90%–100%	90%–100%	90%–100%	90%–100%	90%–100%	90%–100%	90%–100%	90%–100%	90%–100%

SpO_2_ indicates peripheral arterial oxyhaemoglobin saturation.

*Kruskal-Wallis test comparing SpO_2_ by age category; p=0.0387.

†Kruskal-Wallis test comparing SpO_2_ by age category; p=0.0095.

‡Kruskal-Wallis test comparing SpO_2_ by age category; p=0.0401.

SpO_2_, peripheral arterial oxyhaemoglobin saturation.

### SpO_2_ distribution

**Figure 3:**
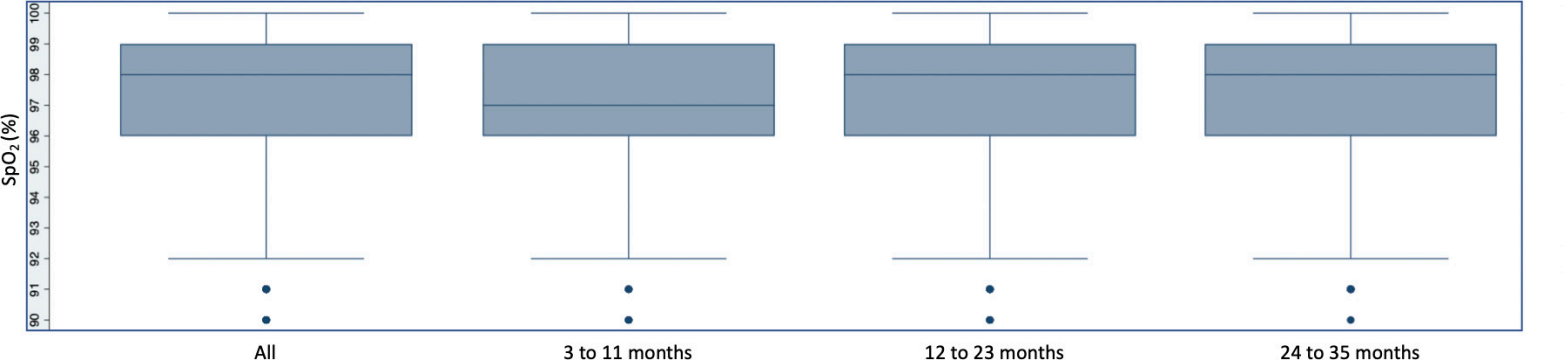
Peripheral arterial oxyhaemoglobin saturation (SpO_2_) distribution in analytical sample 1.

The median SpO_2_ of children included in primary analytical sample 1 was 98% (IQR 96%–99%) and the 10th, 5th and 2.5th percentile SpO_2_ was 94%, 92% and 91% (table 1 and figure 3). Analytical samples 2 and 3 revealed similar findings (table 1 and online supplemental figures 1 and 2). No child included in any of the three analytical samples had a SpO_2_ <90%.

10.1136/bmjresp-2021-001023.supp1 Supplementary data

10.1136/bmjresp-2021-001023.supp2 Supplementary data

### Effects of age and sex on SpO_2_

After stratifying measurements into three age strata, 3–11 months, 12–23 months and 24–35 months, we found children 3–11 months old in primary analytical sample 1 to have a median SpO_2_ of 97%, compared with 98% for each of the two older age strata (p=0.038; table 1 and figure 3). We observed similar findings in analytical samples 2 and 3 (table 1 and online supplemental figures 1 and 2). When regressing SpO_2_ on age in months, adjusted for sex, we found that for every 1 month increase in age the SpO_2_ increased by 0.01% (95% CI 0.001% to 0.02%, p=0.030) in analytical sample 1 (online supplemental figure 3). We did not observe any difference in the SpO_2_ distribution after stratifying by child sex (p=0.959).

10.1136/bmjresp-2021-001023.supp3 Supplementary data

### Health system implications of varying SpO_2_ thresholds

To examine possible consequences on the health system of a SpO_2_ threshold for defining hypoxaemia derived from well children we report the probability of a false positive measurement in table 2 from each analytical sample at differing thresholds. If applying a <92% threshold, for example, 76/1470 (5.1%) well children included in analytical sample 1 would be incorrectly recommended for referral or hospitalisation. SpO_2_ thresholds at ≤90%, <91% and <93% would incorrectly identify 13 (0.8%), 40 (2.7%) and 117 (7.9%) of the 1470 children in analytical sample 1 for hospitalisation, respectively.

**Table 2:** False positive measurements for hypoxaemia by analytical sample and percentile threshold

Analytical sample		2.5th percentile SpO_2_ threshold		5th percentile SpO_2_ threshold		10th percentile SpO_2_ threshold	
	Age in months	SpO_2_	False positive, n/N (%)	SpO_2_	False positive, n/N (%)	SpO_2_	False positive, n/N (%)
1	All	91%	40/1470 (2.7)	92%	76/1470 (5.1)	94%	179/1470 (12.1)
	3–11	92%	20/445 (4.4)	93%	34/445 (7.6)	94%	52/445 (11.6)
	12–23	91%	17/567 (2.9)	92%	35/567 (6.1)	94%	83/567 (14.6)
	24–35	91%	13/458 (2.8)	93%	29/458 (6.3)	95%	72/458 (15.7)
2	All	91%	51/1865 (2.7)	92%	107/1865 (5.7)	94%	248/1865 (13.2)
	3–11	91%	16/615 (2.6)	92%	33/615 (5.3)	94%	85/615 (13.8)
	12–23	91%	21/673 (3.1)	92%	44/673 (6.5)	93%	68/673 (10.1)
	24–35	91%	14/577 (2.4)	92%	30/577 (5.1)	94%	60/577 (10.3)
3	All	91%	55/2022 (2.7)	92%	119/2022 (5.8)	94%	273/2022 (13.5)
	3–11	91%	18/634 (2.8)	92%	35/634 (5.5)	94%	88/634 (13.8)
	12–23	91%	21/698 (3.0)	92%	48/698 (6.8)	93%	72/698 (10.3)
	24–35	92%	36/690 (5.2)	92%	36/690 (5.2)	94%	76/690 (11.0)

SpO_2_, peripheral arterial oxyhaemoglobin saturation.

## Discussion

We derived possible SpO_2_ thresholds for hypoxaemia from the 2.5th, 5th and 10th percentile cutoffs of well children in rural Bangladesh and estimated the probability of false positive measurements assuming a revised threshold was adopted into care. The SpO_2_ threshold is critical as it triggers a cascade of potentially life or death healthcare decisions and understanding the probability of a false positive SpO_2_ measurement for hypoxaemia permits health policy-makers to decide how best to balance mortality risk with anticipated hospitalisation volumes.

There are three key findings from this research. First, cutoffs for hypoxaemia from the 2.5th, 5th and 10th percentile are all higher than the current WHO-defined <90% threshold and we did not find any well children below the SpO_2_ <90% threshold. Thus, if any of these cutoffs for hypoxaemia are adopted then measuring the SpO_2_ earlier in clinical care pathways when healthier children may be over-represented could increase false positive measurements. This has important implications in health systems with limited resources and potential challenges coping with a higher volume of patient referrals. These results, coupled with findings from Malawi that children with LRI and a SpO_2_ between 90% and 92% are at elevated mortality risk, suggest that the current referral threshold of SpO_2_ <90% minimises false positives at the expense of false negatives.^6–8^ In order to ensure minimal misclassification of well children as hypoxaemic, we recommend care algorithms incorporating a hypoxaemia threshold at SpO_2_ levels higher than <90% also consider the child’s clinical status when deciding whether to refer and hospitalise. Second, we found that the SpO_2_ distribution differs by age. Age may, therefore, need to be considered when establishing a SpO_2_ threshold for hypoxaemia. Third, these data show that a 2.5th percentile SpO_2_ threshold in all analytical samples and nearly all age categories is at a 91% cut-off. When compared with the currently recommended <90% WHO cut-off (or <89% inclusive), this <91% threshold (or <91% inclusive) is at the margin of the 2% accuracy range for most pulse oximeters.^19^ As a result, if a higher SpO_2_ threshold were to be considered the 5th or 10th percentile threshold may be more suitable when considering inherent device accuracy limitations. Overall, future analyses that include unwell children should consider prioritising SpO_2_ thresholds outside of the device accuracy range, such as the 5th or 10th percentile in this analysis, as well as whether the statistical relationship between age and SpO_2_ distribution has clinical significance.

To date, there have been few attempts to establish a reference range for SpO_2_ measurements of children in LMICs living at lower altitudes. Since most children reside at lower altitudes, understanding the SpO_2_ distribution among this population has broad relevance for identifying the optimal SpO_2_ threshold for hypoxaemia. In one study from Chennai, India (altitude 7 m) the authors measured the SpO_2_ of 626 healthy children aged 1 month to 5 years.^20^ In contrast to our findings, the authors found no difference in the SpO_2_ distribution by age. The inclusive fifth percentile of participants in Chennai was a SpO_2_ <96%, which was 4% higher than in our study. Other studies that also included healthy children from lower altitudes reported the mean and SD of the SpO_2_ distribution.^13 14 21^ Given the SpO_2_ distribution of healthy children is negatively skewed we described these data using median and percentiles and are therefore unable to make meaningful comparisons.

A more recent multicountry study from the Household Air Pollution Intervention Network (HAPIN) investigators evaluated the SpO_2_ distribution among 1134 healthy children <24 months old from three lower altitude settings including the same region of India as the prior study (Nagapattinam (altitude 9 m) and Villupuram (altitude 44 m), and also in Guatemala (Jalapa District, altitude 1417 m) and Rwanda (Kayonza District, altitude 1354 m) and one high altitude setting in Puno, Peru (altitude 3827 m).^22^ The fifth percentile threshold reported by HAPIN investigators in India was notably consistent with the Chennai study at 96%. Unlike the Chennai study, however, HAPIN investigators found lower fifth percentile thresholds for age in Rwanda (92%) and Guatemala (93%), and observed a correlation between younger age and lower SpO_2_. None of these studies reported the 2.5th percentile cut-off. Overall, it is somewhat surprising that our data from Bangladesh aligns closer with Rwanda and Guatemala than India, another South Asian setting closer in altitude.

Methodology may largely be responsible for the variation in results across these studies. Variation may be due to a combination of device accuracy, including differences in accuracy between devices, variation inherent to measurements on children, measurement variation between healthcare workers and healthcare worker cadres with different training backgrounds, and possible varying degrees of misclassification bias of sick children in each of the three studies. Specifically, the Chennai study used a different pulse oximeter (L&T Medical, Stellar P) than in the HAPIN study and our study, which both used Masimo devices (Rad-97 and Rad-5).^20 22^ Pulse oximeter SpO_2_ estimation algorithms are known to differ by manufacturer and in the US the Food and Drug Administration requires the testing and certification of pulse oximeters to be accurate within a root mean square error of 3% for arterial blood saturation values between 70% and 100%.^19^ Although data comparing pulse oximeter device performance in children in LMICs is notably limited, it is also well known that device performance can change under conditions common to children like motion and low perfusion.^23 24^

A key methodological difference in this study, compared with the Chennai and HAPIN studies, was formal healthcare workers at healthcare facilities conducting recruitment. In our study, trained but informal CHWs recruited and screened children within the community. Although we employed intensive efforts to train and supervise CHWs our use of informal healthcare workers may have influenced both pulse oximeter measurement quality and the number of unwell children remaining in our sample. Our post hoc data cleaning attempted to further address these possible weaknesses and our findings are reassuringly consistent across the three analytical samples. By contrast, the Chennai and HAPIN studies did not further restrict their analyses by heart rate reference ranges, and therefore, could remain vulnerable to these issues.

Lastly, although not explicitly stated in either of the Chennai or HAPIN studies, we intentionally did not exclude children with isolated nasal congestion and/or rhinorrhoea as anecdotally they are common to otherwise healthy rural Bangladeshi children. In Bangladesh, both indoor and ambient air pollution is marked and includes environmental irritants that can cause ongoing upper respiratory mucosal inflammation, nasal congestion and/or rhinorrhoea typical of non-allergic, non-infectious rhinitis.^25^ While it is unlikely that any misclassification bias or device performance inconsistencies were substantially different in our study than in the Chennai and HAPIN studies, it is nevertheless important to interpret our results within this context.

Another issue that may impact the utility of pulse oximeters in all LMICs is the possible greater inaccuracy of SpO_2_ measurements in individuals with darker skin pigmentation.^26^ Although pulse oximetry inaccuracy in individuals with darker skin pigmentation is well established, a recent publication of hospitalised adults in the USA suggests the magnitude and direction of SpO_2_ measurement bias in people with darker skin tones may be clinically unacceptable.^26^ The authors reported a higher odds of hypoxaemia—as measured by an arterial blood gas—among darkly pigmented adults with a normal SpO_2_, compared with adults without dark skin pigmentation and a normal SpO_2_.^26^ Further study is needed to better understand the relative contribution and direction of pulse oximeter inaccuracy among children in LMICs where higher percentages of the population often have darker skin pigmentation.

In sum, our findings provide possible reference SpO_2_ thresholds for hypoxaemia derived from a population of well children in Bangladesh residing at lower altitude. In fragile, overburdened health systems higher false positive measurements may limit the implementation feasibility of SpO_2_ thresholds above the current <90% mark without additional strengthening of clinical assessments by healthcare providers. This research suggests that age needs to be considered in further work on establishing thresholds for hypoxaemia. Key next steps include determining the mortality risk of ill children with SpO_2_ measurements at or below these thresholds in varying LMICs, including Bangladesh, as well as evaluating the performance of SpO_2_ at or below these thresholds for diagnosing LRI. Such research will also shed light on the potential mortality implications of false negative measurements when applying a lower <90% SpO_2_ threshold for hypoxaemia.

## Data Availability

Data are available on reasonable request.

## References

[1] GBD 2017 Lower Respiratory Infections Collaborators. Quantifying risks and interventions that have affected the burden of lower respiratory infections among children younger than 5 years: an analysis for the global burden of disease study 2017. Lancet Infect Dis 2020;20:60–79. 10.1016/S1473-3099(19)30410-4 31678026 PMC7185492

[2] McCollum ED, Ginsburg AS. Outpatient management of children with World Health Organization chest indrawing pneumonia: implementation risks and proposed solutions. Clin Infect Dis 2017;65:1560–4. 10.1093/cid/cix543 29020216 PMC5850637

[3] Lazzerini M, Sonego M, Pellegrin MC. Hypoxaemia as a mortality risk factor in acute lower respiratory infections in children in low and middle-income countries: systematic review and meta-analysis. PLoS One 2015;10:e0136166. 10.1371/journal.pone.0136166 26372640 PMC4570717

[4] World Health Organization. Integrated management of childhood illness: chart booklet. Geneva, Switzerland: World Health Organization, 2014. https://apps.who.int/iris/bitstream/handle/10665/104772/9789241506823_Chartbook_eng.pdf

[5] World Health Organization,. Pocketbook of hospital care for children. guidelines for the management of common childhood illnesses. second edition. 2nd edn. Geneva, Switzerland: World Health Organization, 2013. https://apps.who.int/iris/bitstream/handle/10665/81170/9789241548373_eng.pdf?sequence=1

[6] Hooli S, Colbourn T, Lufesi N, et al. Predicting hospitalised paediatric pneumonia mortality risk: an external validation of RISC and mRISC, and local tool development (RISC-Malawi) from Malawi. PLoS One 2016;11:e0168126. 10.1371/journal.pone.0168126 28030608 PMC5193399

[7] Colbourn T, King C, Beard J, et al. Predictive value of pulse oximetry for mortality in infants and children presenting to primary care with clinical pneumonia in rural Malawi: a data linkage study. PLoS Med 2020;17:e1003300. 10.1371/journal.pmed.1003300 33095763 PMC7584207

[8] Hooli S, King C, Zadutsa B, et al. The epidemiology of hypoxemic pneumonia among young infants in Malawi. Am J Trop Med Hyg 2020;102:676–83. 10.4269/ajtmh.19-0516 31971153 PMC7056410

[9] Gray D, Willemse L, Visagie A, et al. Lung function and exhaled nitric oxide in healthy unsedated African infants. Respirology 2015;20:1108–14. 10.1111/resp.12579 26134556 PMC4623783

[10] Sachdev HS, Porwal A, Acharya R, et al. Haemoglobin thresholds to define anaemia in a national sample of healthy children and adolescents aged 1-19 years in India: a population-based study. Lancet Glob Health 2021;9:e822–31. 10.1016/S2214-109X(21)00077-2 33872581 PMC7612991

[11] Subhi R, Smith K, Duke T. When should oxygen be given to children at high altitude? A systematic review to define altitude-specific hypoxaemia. Arch Dis Child 2009;94:6–10. 10.1136/adc.2008.138362 18829620

[12] Lozano JM, Steinhoff M, Ruiz JG, et al. Clinical predictors of acute radiological pneumonia and hypoxaemia at high altitude. Arch Dis Child 1994;71:323–7. 10.1136/adc.71.4.323 7979525 PMC1030011

[13] Reuland DS, Steinhoff MC, Gilman RH, et al. Prevalence and prediction of hypoxemia in children with respiratory infections in the Peruvian Andes. J Pediatr 1991;119:900–6. 10.1016/S0022-3476(05)83040-9 1960604

[14] Onyango FE, Steinhoff MC, Wafula EM, et al. Hypoxaemia in young Kenyan children with acute lower respiratory infection. BMJ 1993;306:612–5. 10.1136/bmj.306.6878.612 8369033 PMC1676956

[15] Andrade V, Andrade F, Riofrio P, et al. Pulse oximetry curves in healthy children living at moderate altitude: a cross-sectional study from the Ecuadorian Andes. BMC Pediatr 2020;20:440. 10.1186/s12887-020-02334-z 32948159 PMC7499919

[16] McCollum ED, Ahmed S, Roy AD, et al. Effectiveness of the 10-valent pneumococcal conjugate vaccine against radiographic pneumonia among children in rural Bangladesh: a case-control study. Vaccine 2020;38:6508–16. 10.1016/j.vaccine.2020.08.035 32873404 PMC7520553

[17] Baqui AH, McCollum ED, Saha SK, et al. Pneumococcal conjugate vaccine impact assessment in Bangladesh. Gates Open Res 2018;2:21. 10.12688/gatesopenres.12805.1 29984359 PMC6030398

[18] Fleming S, Thompson M, Stevens R, et al. Normal ranges of heart rate and respiratory rate in children from birth to 18 years of age: a systematic review of observational studies. Lancet 2011;377:1011–8. 10.1016/S0140-6736(10)62226-X 21411136 PMC3789232

[19] ISO 80601-2-61:2017. Medical electrical equipment — part 2-61: particular requirements for basic safety and essential performance of pulse oximeter equipment. Available: https://www.iso.org/standard/67963.htmlBalasubramanian [Accessed Jun 2021].

[20] Balasubramanian S, Suresh N, Ravichandran C, et al. Reference values for oxygen saturation by pulse oximetry in healthy children at sea level in Chennai. Ann Trop Paediatr 2006;26:95–9. 10.1179/146532806X107421 16709326

[21] Madico G, Gilman RH, Jabra A, et al. The role of pulse oximetry. its use as an indicator of severe respiratory disease in Peruvian children living at sea level. respiratory group in Peru. Arch Pediatr Adolesc Med 1995;149:1259–63. 10.1001/archpedi.1995.02170240077012 7581759

[22] Crocker ME, Hossen S, Goodman D, et al. Effects of high altitude on respiratory rate and oxygen saturation reference values in healthy infants and children younger than 2 years in four countries: a cross-sectional study. Lancet Glob Health 2020;8:e362–73. 10.1016/S2214-109X(19)30543-1 32087173 PMC7034060

[23] Lipnick MS, Feiner JR, Au P, et al. The accuracy of 6 inexpensive pulse oximeters not cleared by the food and drug administration: the possible global public health implications. Anesth Analg 2016;123:338–45. 10.1213/ANE.0000000000001300 27089002

[24] Louie A, Feiner JR, Bickler PE, et al. Four types of pulse oximeters accurately detect hypoxia during low perfusion and motion. Anesthesiology 2018;128:520–30. 10.1097/ALN.0000000000002002 29200008

[25] Bousquet J, Anto JM, Annesi-Maesano I, et al. POLLAR: impact of air pollution on asthma and rhinitis; a European Institute of innovation and technology health (EIT health) project. Clin Transl Allergy 2018;8:36. 10.1186/s13601-018-0221-z 30237869 PMC6139902

[26] Sjoding MW, Dickson RP, Iwashyna TJ, et al. Racial bias in pulse oximetry measurement. N Engl J Med 2020;383:2477–8. 10.1056/NEJMc2029240 33326721 PMC7808260

