# Supplementary figures and images for: Defining hypoxaemia from pulse oximeter measurements of oxygen saturation in well children at low altitude in Bangladesh: an observational study
Source: BMJ Open Respir Res. 2021 Nov 2;8(1):e001023. doi: 10.1136/bmjresp-2021-001023 (PMC8565559; doi:10.1136/bmjresp-2021-001023)

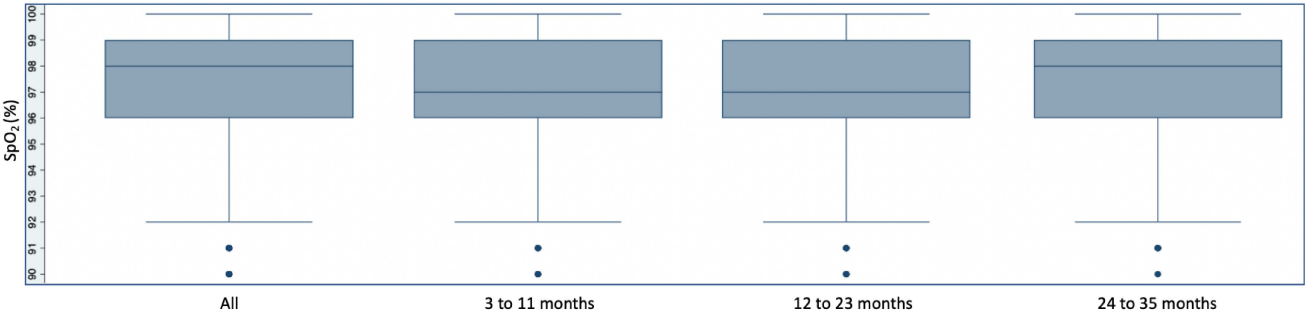

Supplement: Supplementary data [file bmjresp-2021-001023supp001.pdf]

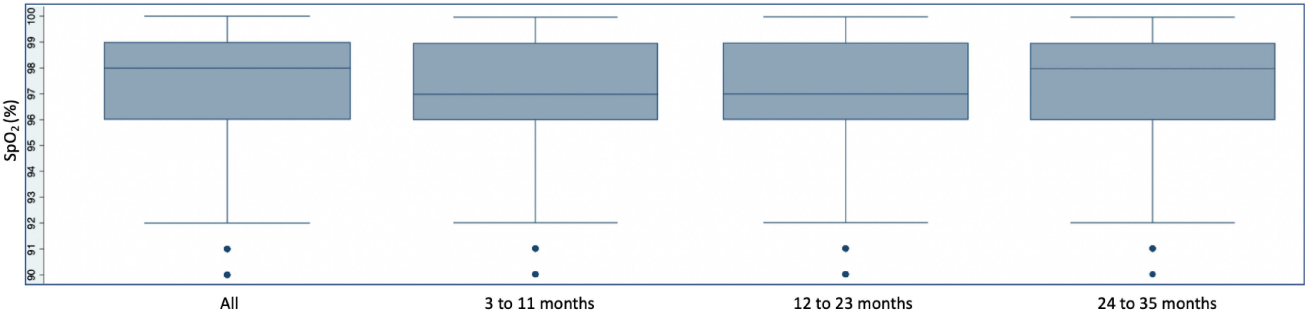

Supplement: Supplementary data [file bmjresp-2021-001023supp002.pdf]

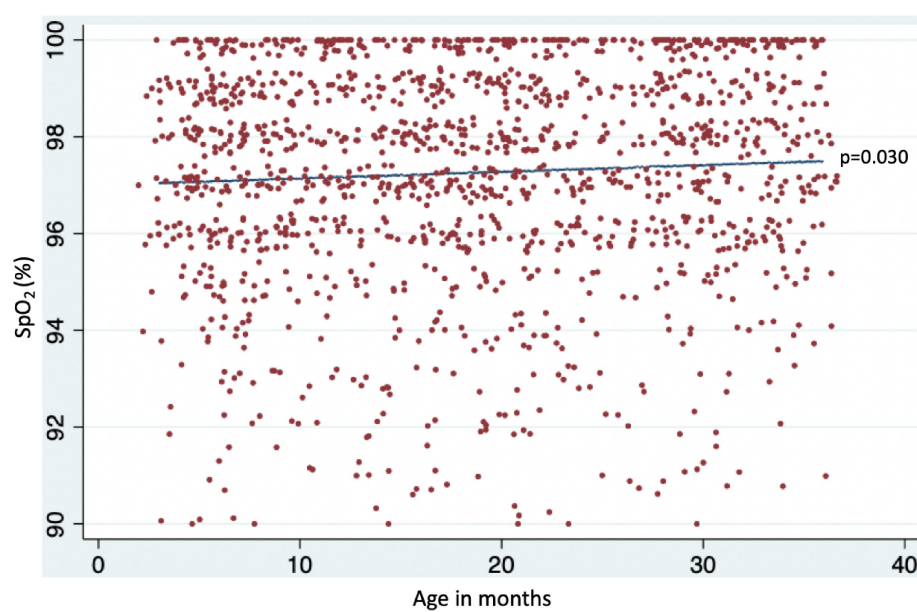

Supplement: Supplementary data [file bmjresp-2021-001023supp003.pdf]
